# Nox2-derived ROS in PPARγ signaling and cell-cycle progression of lung alveolar epithelial cells

**DOI:** 10.1016/j.freeradbiomed.2011.05.027

**Published:** 2011-08-01

**Authors:** Jennifer Tickner, Lampson M. Fan, Junjie Du, Daniel Meijles, Jian-Mei Li

**Affiliations:** Faculty of Health and Medical Sciences, University of Surrey, Guildford, Surrey GU2 7XH, UK

**Keywords:** ROS, reactive oxygen species, PPARγ, peroxisome proliferator-activated receptor γ, KO, knockout, DHE, dihydroethidium, BADGE, bisphenol A diglycidyl ether, L-NAME, *N*^ω^-nitro-l-arginine methyl ester, DPI, diphenyleneiodonium, SOD, superoxide dismutase, DDC, diethyldithiocarbamate, MAPK, mitogen-activated protein kinase, NADPH, nicotinamide adenine dinucleotide phosphate, Nox, NADPH oxidase, PCNA, proliferating cell nuclear antigen, DMEM, Dulbecco's modified Eagle medium, Redox signaling, Lung, PPARγ, Nox2, MAPK, Cell cycle, Free radicals

## Abstract

Reactive oxygen species (ROS) play important roles in peroxisome proliferator-activated receptor γ (PPARγ) signaling and cell-cycle regulation. However, the PPARγ redox-signaling pathways in lung alveolar epithelial cells remain unclear. In this study, we investigated the in vivo and in vitro effects of PPARγ activation on the levels of lung ROS production and cell-cycle progression using C57BL/6J wild-type and Nox2 knockout mice (*n* = 10) after intraperitoneal injection of a selective PPARγ agonist (GW1929, 5 mg/kg body wt, daily) for 14 days. Compared to vehicle-treated mice, GW1929 increased significantly the levels of ROS production in wild-type lungs, and this was accompanied by significant up-regulation of PPARγ, Nox2, PCNA, and cyclin D1 and phosphorylation of ERK1/2 and p38MAPK. These effects were absent in Nox2 knockout mice. In cultured alveolar epithelial cells, GW1929 (5 μM for 24 h) increased ROS production and promoted cell-cycle progression from G0/G1 into S and G2/M phases, and these effects were abolished by (1) adding a PPARγ antagonist (BADGE, 1 μM), (2) knockdown of PPARγ using siRNA, or (3) knockout of Nox2. In conclusion, PPARγ activation through Nox2-derived ROS promotes cell-cycle progression in normal mouse lungs and in cultured normal alveolar epithelial cells.

The peroxisome proliferator-activated receptor γ (PPARγ) is one of the three PPARs (α, γ, and β/δ), which belong to the nuclear receptor family of ligand-activated transcription factors. When activated by appropriate ligands, such as prostaglandins, polyunsaturated fatty acids, and leukotrienes, the PPARs bind to *cis*-retinoic acid receptors to form a heterodimer, which then binds to the specific regions on the target genes called peroxisome proliferator hormone-responsive elements (PPRE) to promote or inhibit gene transcription [1]. Among the PPARs, PPARγ activation has been found to have pleiotropic beneficial effects in multiple tissues on lipid metabolism, glucose homeostasis, and cell-cycle regulation [2]. PPARγ ligands have been used routinely for the treatment of type 2 diabetes and are generally well tolerated. Despite proven therapeutic benefits, there are side effects, which include weight gain, fluid retention, potential risk of cardiac failure, and carcinogenicity [3–5].

PPARγ is expressed in almost every cell type of the human body and plays important roles in organ development and function [2,3]. In the lung, PPARγ has been found to play a central role in the early stage of lung development [6]. Mice deficient in PPARγ developed air-space enlargement with decreased tissue resistance and increased lung volume [7], whereas treatment of mice with an agonist to PPARγ protects newborn lungs from hypoxia-induced lung injury [8]. PPARγ deficiency is also linked to pulmonary disorders such as asthma, lung cancer, cystic fibrosis, and inflammation, and PPARγ has been suggested to be a potential therapeutic target to treat these diseases [2]. On the other hand, chronic metabolic and hormonal disturbances are characteristic of type 2 diabetic patients, which predisposes these patients to the risk of lung cancer [9]. Although recently a clinical retrospective database analysis had reported that PPARγ agonists (thiazolidinediones) reduced lung cancer risk in diabetic patients [10], there was no rational explanation and the mechanistic data explaining PPARγ action in lung cancer are controversial. Despite the fact that the majority of type 2 diabetic patients, when starting PPARγ agonist treatment, are middle-aged without lung problems, detailed investigation into the long-term effects of administration of exogenous PPARγ agonists on the healthy lungs of middle-aged individuals is still missing.

The lung epithelial cells also express abundantly an NADPH oxidase (Nox) that generates low levels of ROS under physiological conditions and contributes to the redox regulation of normal lung function [11]. However, the activity of Nox can be up-regulated by various stimuli such as metabolic disorders, and enhanced ROS production contributes to the pathogenesis of lung diseases, such as acute respiratory distress syndrome, bronchopulmonary dysplasia, emphysema, idiopathic pulmonary fibrosis, and cancer [11]. The Nox enzyme has at least seven isoforms, i.e., Nox1–5, Duox1, and Duox2. Among these Nox's, Nox2, also called gp91^phox^, requires the presence of p22^phox^ and regulatory subunits (p40^phox^, p47^phox^, p67^phox^, and rac1) to generate O_2_^•−^. Nox2 has been found to be robustly expressed in lung alveolar epithelial cells and involved in the regulation of epithelial cell function [12].

Although both PPARγ and Nox2 are expressed in the alveolar epithelial cells and play important roles in lung biology, little is known regarding the role of PPARγ in the regulation of lung ROS production by Nox2 or in the redox regulation of lung function. In this study, we investigated the effect of PPARγ activation by GW1929, a high-affinity nonthiazolidinedione PPARγ activator [13], on the levels of ROS production, Nox2 signaling, and cell-cycle progression in the lungs of wild-type and Nox2 knockout (KO) mice, as well as in cultured alveolar epithelial cells isolated from these mice.

## Materials and methods

### Reagents

Dihydroethidium (DHE) was purchased from Molecular Probes (UK). The polyclonal antibodies against p22^phox^, Nox2, Nox4, and α-tubulin were from Santa Cruz Biotechnology; antibodies to phospho-ERK1/2, phospho-p38MAPK, phospho-JNK, proliferating cell nuclear antigen (PCNA), and cyclin D1 were from Cell Signaling Technology. Polyethyleneimine (linear MW 25,000) was from Polysciences (UK). GW1929 and other reagents and chemicals were from Sigma unless stated otherwise.

### Animals and GW1929 treatment.

Wild-type and Nox2 KO mice on the C57BL/6 background (The Jackson Laboratory) were bred in our institution from heterozygous mice and genotyped. All studies were performed in accordance with protocols approved by the Home Office under the Animals (Scientific Procedures) Act 1986 UK. Animals were housed under standard conditions with a 12-h light and dark cycle and fed a normal chow diet. Middle-aged male mice (6–7 months of age, *n* = 10–12/per group) were injected intraperitoneally once daily with either vehicle (4% dimethyl sulfoxide (DMSO)/phosphate-buffered saline (PBS)) or GW1929 (5 mg/kg body wt in 4% DMSO/PBS). The dose was chosen based on previous publications [13,14]. Measurements of blood pressure before and after treatment were obtained using computerized tail-cuff plethysmography in conscious mice after 5 days of training periods. On day 14, the levels of fasting glucose were taken and the levels of fasting serum insulin were measured using a mouse insulin ELISA kit (Mercodia Developing Diagnostic, Sweden). Mice were killed and body weights were obtained. Tissues were harvested, washed, and snap-frozen for further experiments.

### Cell culture

Pulmonary alveolar epithelial cells of wild-type and Nox2 KO mice were obtained by primary explant culture. Briefly, blocks of pulmonary alveolar tissues (2 mm^3^ in dimension) of untreated animals were seeded onto cell culture dishes and incubated at 37 °C, 5% CO_2_, in 10% fetal calf serum (FCS)/DMEM. After 2 weeks of culture, outgrowing alveolar epithelial cells were characterized by flow cytometry. Cells were counted and reseeded at 1 × 10^6^/100-mm^2^ dish and cultured for 24 h to reach 95% confluence. Cells were then treated with either vehicle (0.125% DMSO) or GW1929 (5 μM) or bisphenol A diglycidyl ether (BADGE; 1 μM) in culture medium for 48 h before being harvested and used for further analysis.

### Measurement of ROS production

Tissue homogenates were prepared as described previously [15]. O_2_^•−^ production by tissue homogenates was measured by lucigenin chemiluminescence (Lumistar; BMG) as described previously [15]. The specificity of O_2_^•−^ thus measured was confirmed by adding tiron (10 mM), a nonenzymatic scavenger of O_2_^•−^. The enzymatic sources of O_2_^•−^ production were identified by preincubation of tissue homogenates with inhibitors targeting nitric oxide synthase (*N*^ω^-nitro-l-arginine methyl ester (L-NAME), 100 μM), the mitochondrial electron transport chain (rotenone, 50 μM), xanthine oxidase (oxypurinol, 250 μM), flavo-proteins (diphenyleneiodonium (DPI), 20 μM), and Nox (apocynin, 20 μM). The effect of Cu/Zn superoxide dismutase (SOD) on ROS production was investigated by preincubation (30 min) of tissue homogenates with a specific inhibitor, diethyldithiocarbamate (DDC; 200 μM) before O_2_^•−^ measurement. ROS generation within the lung tissues in situ was also measured using DHE fluorescence as described previously [16]. Fluorescence intensity was acquired using a Zeiss LS510 confocal microscopy system and quantified from at least three random fields (269.7 × 269.2 μm) per slide, three slides per animal, and six animals per group.

### Immunoblotting

Soluble protein concentrations of the lung homogenate were determined by using a Bio-Rad kit (Bio-Rad Laboratories, UK). Immunoblotting using 40 μg protein per lane was performed as described previously [16]. The images were captured digitally using a BioSpectrum AC imaging system (UVP, UK), and the densities of the protein bands were normalized to the loading control bands and quantified. The results were expressed as the means ± SD of six animals.

### Immunofluorescence microscopy

Sample preparation and confocal microscopy were performed as described previously [16]. Briefly, frozen sections (6 μm) were firstly treated with a biotin blocking kit (DAKO) according to the manufacturer's instructions. Primary antibodies were used at a 1:250 dilution in PBS with 0.1% bovine serum albumin for 30 min at room temperature. Biotin-conjugated anti-rabbit or anti-goat (1:1000 dilution) antibodies were used as secondary antibodies. Specific binding was detected by extravidin–FITC or streptavidin–Cy3. Normal rabbit or goat IgG (5 μg/ml) was used instead of primary antibody as a negative control. Images were acquired on an Olympus BX61 fluorescence microscopy system and stored digitally for analysis. Fluorescence intensities of samples acquired under the same microscopic parameter were quantified from at least three random fields (433 × 330 μm) per slide, three slides per animal, and six animals per group.

### PPARγ or Nox2 small interfering RNA (siRNA) transfection

Primary pulmonary alveolar epithelial cells were plated (5 × 10^5^ cells/well) in six-well plates in 10% FCS/DMEM. Mouse PPARγ siRNA and a random scrambled control siRNA were from Santa Cruz Biotechnology (sc-29456). The nucleotide sequences of Nox2 siRNA and a random negative control siRNA were as described previously [16]. The gene transfection was performed as described previously [16], with some modification. Briefly, 3 μg siRNA was mixed with 30 μl polyethyleneimine (concentration 1 mg/ml) and then diluted in 300 μl of serum-free DMEM. The transfection mixture was incubated at room temperature for 15 min. Cells were washed and the medium was replaced with 1.2 ml serum-free DMEM per well. The transfection mixture (300 μl) was added into the cell culture to make up to 1.5 ml/well. Cells were incubated for 4 h before 1.5 ml of 20% FCS/DMEM was added into the well. Cells were cultured continually overnight. The next morning, the medium was replaced with fresh 10% FCS/DMEM. After 24 h of culture, cells were treated with either vehicle or 5 μM GW1929 for 48 h as described above.

### Cell cycle analysis

After 48 h of treatment with vehicle or GW1929, the cells were detached with trypsin/EDTA, counted, and used immediately for cell-cycle analysis as described previously [17]. Briefly, the cells were fixed with 70% ethanol, treated with RNase A (0.2 mg/ml), and then stained with propidium iodide (50 μg/ml) for DNA content. The number of cells in G0/G1, S, and G2/M phases and of apoptotic cells was counted using an Accuri C6 flow cytometer and analyzed using the Cflow program.

### Statistical analysis

Data are presented as means ± SD of the results from 10–12 mice per group for the in vivo experiments, and from at least three independent cell isolations for the in vitro experiments. Comparisons were made by unpaired Student's *t* test, with Bonferroni correction for multiple testing. *P* < 0.05 was considered statistically significant.

## Results

### The effects of GW1929 on lung ROS production

**Fig. 1:**
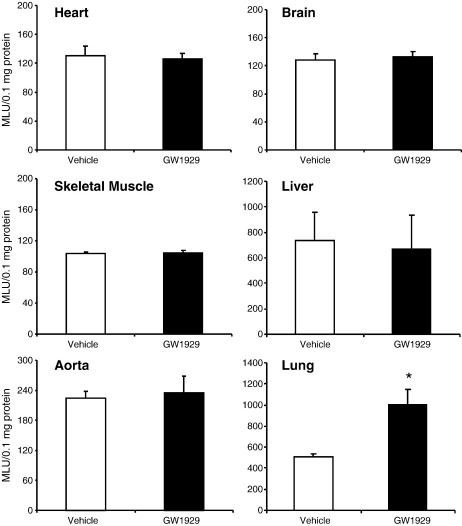
The effects of in vivo treatment with GW1929 on NADPH-dependent O_2_^•−^ production by various organ homogenates measured by lucigenin chemiluminescence. *P < 0.05 for indicated values versus vehicle values in the same organ.

Compared to vehicle-treated controls, treatment of both wild-type and Nox2 KO mice (6–7 months of age) with GW1929 (5 mg/kg body wt, daily) for 14 days had no significant effect on the body weight, blood pressure, fasting blood glucose, and insulin levels (Table 1). We then examined the potential effects of systemic administration of GW1929 on NADPH-dependent O_2_^•−^ production in various organs of wild-type mice using lucigenin (5 μM) chemiluminescence (Fig. 1). There was no significant difference in the levels of O_2_^•−^ production in the hearts, skeletal muscles, aortas, brains, or livers between mice treated with GW1929 and those treated with vehicle. However, there was an ~ 2.5-fold increase in the levels of O_2_^•−^ production in the lungs of GW1929-treated mice compared to vehicle-treated controls.

**Fig. 2:**
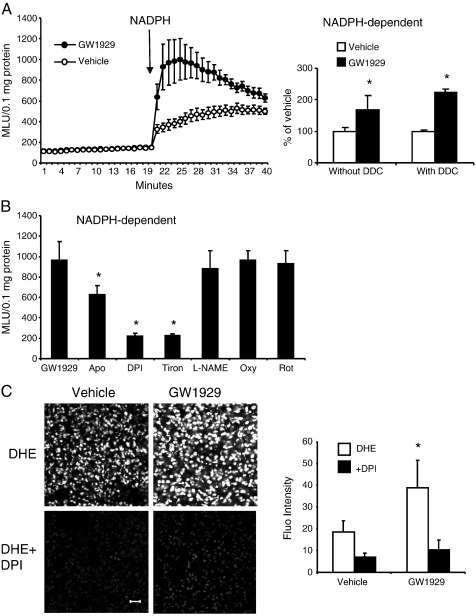
The effects of in vivo treatment with GW1929 on ROS production by wild-type mouse lungs. (A) Lucigenin chemiluminescence using lung homogenates. Left: kinetic measurement of O_2_^•−^ production. Right: the effect of Cu/Zn-SOD inhibitor, DDC, on the levels of NADPH-dependent O_2_^•−^ production. *P < 0.05 for indicated values versus vehicle values. (B) The effects of various enzyme inhibitors on the levels of NADPH-dependent O_2_^•−^ production measured by lucigenin chemiluminescence. *P < 0.05 for indicated values versus GW1929 values. (C) Left: representative images of DHE fluorescence in lung sections. Right: quantification of DHE fluorescence. *P < 0.05 for indicated values versus vehicle values under the same conditions. Scale bar = 50 μm.

The effect of GW1929 on wild-type lung ROS production was further examined in detail (Fig. 2). In the absence of NADPH, the basic levels of lung O_2_^•−^ production were very low and there was no significant difference between vehicle- and GW1929-treated groups. However, when NADPH was added, there were significant increases in the levels of O_2_^•−^ production by GW1929-treated lungs compared to vehicle-treated controls (Fig. 2A, left). Increased NADPH-dependent O_2_^•−^ production found in GW1929-treated lungs was not due to the changes in the expression and activity of the extracellular Cu/Zn SOD, because preincubation of lung homogenates with a Cu/Zn SOD inhibitor, DDC (200 μM), for 30 min had no significant effect on the levels of ROS production compared to samples without DDC pretreatment (Fig. 2A, right). GW1929-induced increase in O_2_^•−^ production was inhibited by apocynin (a Nox inhibitor) or DPI (a flavo-protein inhibitor), but not by L-NAME (NOS inhibitor), oxypurinol (xanthine oxidase inhibitor), or rotenone (mitochondrial complex 1 enzyme inhibitor) (Fig. 2B). Tiron, a specific O_2_^•−^ scavenger, was used to confirm the detection of O_2_^•−^ by chemiluminescence. The GW1929-induced lung ROS production was further examined in situ by DHE fluorescence on lung sections (Fig. 2C). Significant increases in DHE fluorescence were observed in GW1929-treated lungs compared to the vehicle controls. Moreover, the GW1929 effect was significantly inhibited in the presence of DPI. Put together, these results suggested that Nox might be responsible for increased lung O_2_^•−^ production after GW1929 treatment.

**Fig. 3:**
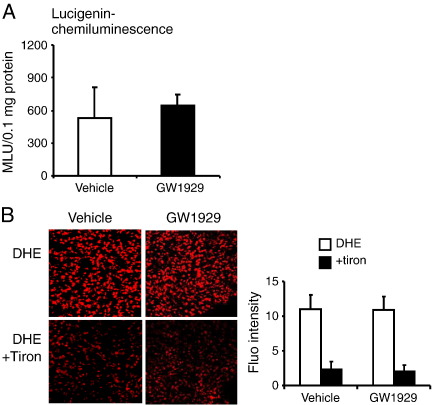
The effects of in vivo treatment with GW1929 on ROS production by Nox2 KO lungs. (A) NADPH-dependent lucigenin chemiluminescence. (B) DHE fluorescence; tiron was used to confirm the detection of O_2_^•−^.

We then treated the Nox2 KO mice with GW1929 for 14 days and measured the NADPH-dependent lung O_2_^•−^ production exactly as we did for the wild-type mice. Compared to vehicle-treated wild-type mice, GW1929 treatment had no significant effect on the levels of O_2_^•−^ production by Nox2 KO lungs as examined by lucigenin chemiluminescence (Fig. 3A) or by DHE fluorescence (Fig. 3B).

### The effects of GW1929 on lung expression of PPARγ and Nox

**Fig. 4:**
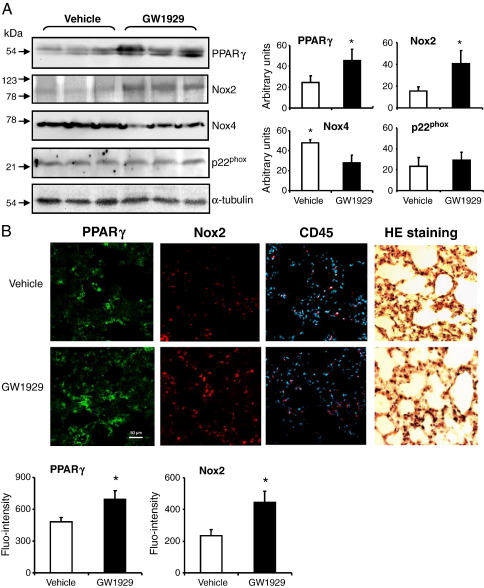
The effects of GW1929 on lung expression of PPARγ and Nox. (A) Western blot. Protein bands were quantified and normalized to the levels of the α-tubulin detected in the same samples. (B) Immunofluorescence. PPARγ was labeled by FITC (green), and Nox2 was labeled by Cy3 (red). CD45 was labeled by Cy3 (red) and the nuclei were labeled by DAPI (blue) to show the total cell numbers. Hematoxylin and eosin staining showed the lung morphology. *P < 0.05 for indicated values versus vehicle values. values.

The results of lung O_2_^•−^ production strongly suggested an involvement of Nox2 enzyme; we therefore examined the levels of protein expression of PPARγ, Nox2, Nox4, and p22^phox^ in the wild-type lungs by Western blotting (Fig. 4A). Compared to vehicle-treated controls, GW1929 increased significantly the protein expression of PPARγ and Nox2, and this was coupled to a decrease in Nox4 expression. There was no significant change in the levels of p22^phox^ expression. The increases in the expression of PPARγ (FITC, green) and Nox2 (Cy3, red) were further confirmed by immunofluorescence (Fig. 4B). There was no significant difference in the numbers of CD45^+^ cells (Cy3, red) between vehicle- and GW1929-treated lung sections as counted against the total cell nuclei labeled with DAPI (blue). Parallel sections were stained with hematoxylin and eosin to show the lung morphology.

### GW1929-induced MAPK activation and cell-cycle molecule expression in the lungs

**Fig. 5:**
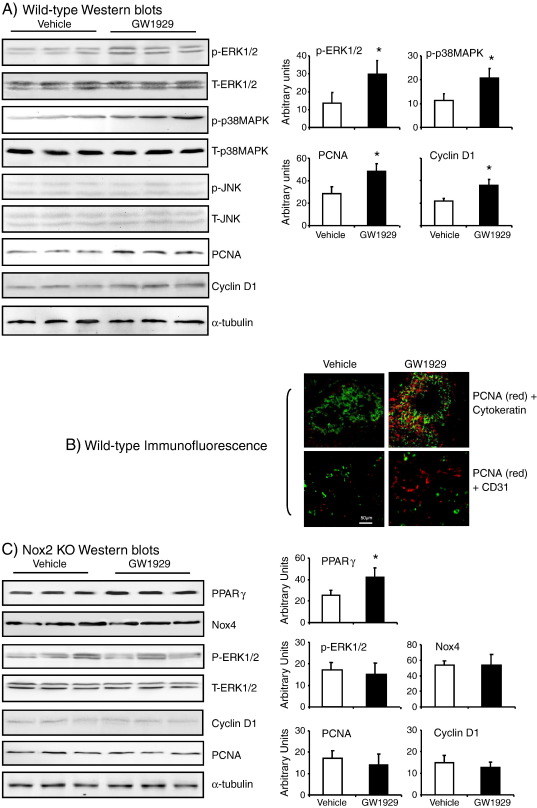
MAPK phosphorylation and the expression of PCNA and cyclin D1 in the lung tissues. (A) Western blots of lung homogenates. (B) Immunofluorescence of lung sections. PCNA was labeled by Cy3 (red) and cytokeratin or CD31 was labeled by FITC (green). (C) Western blots of Nox2 KO lung homogenates. The levels of the protein bands detected by Western blot were quantified and normalized to the levels of α-tubulin detected in the same sample. *P < 0.05 for indicated values versus vehicle values in the same graph.

The mitogen-activated protein kinases (MARKs) have been reported to be redox sensitive and involved in Nox2 signaling [16]. MAPKs are also important downstream signaling molecules of PPARγ [18]. To find out whether MAPKs are involved in bridging PPARγ and Nox2 signaling, we examined the phosphorylation of ERK1/2, p38MAPK, and JNK in the wild-type lungs by immunoblotting using phosphorylation-specific monoclonal antibodies (Fig. 5A). There was no significant change in the expression of total ERK1/2 and p38MAPK between vehicle- and GW1929-treated groups. However, there were significant increases in the levels of ERK1/2 and p38MAPK phosphorylation in the GW1929-treated group compared to vehicle-treated controls. The levels of JNK expression were very low without a significant difference between vehicle- and GW1929-treated groups.

Since MAPKs are upstream signaling molecules involved in cell-cycle regulation, we examined also the levels of expression of PCNA and cyclin D1, two of the critical molecules required for cell proliferation and cell-cycle progression from G0/G1 phase to S phase. We found that GW1929 treatment increased significantly the protein expression of PCNA and cyclin D1 in the wild-type lungs compared to vehicle-treated controls (Fig. 5A). The levels of α-tubulin in the same samples were used as loading controls. Immunofluorescence double staining of lung sections with PCNA (red) plus cytokeratin (epithelial cell marker, green) or CD31 (endothelial cell marker, green) indicated that increased expression of PCNA was in the alveolar epithelial cells (Fig. 5B).

We then examined the expression of PPARγ, Nox4, PCNA, and cyclin D1 and the phosphorylation of ERK1/2 in the Nox2 KO lungs treated with vehicle or GW1929 (Fig. 5C). We found that GW1929 treatment increased significantly the lung expression of PPARγ in Nox2 KO mice. However, in the absence of Nox2, GW1929 had no significant effect on the levels of expressions of Nox4, PCNA, and cyclin D1. Similarly, there was no significant difference in the levels of ERK1/2 phosphorylation between vehicle- and GW1929-treated lungs. The levels of expression of α-tubulin in the same samples were used as loading controls.

### GW1929-induced ROS production and cell-cycle progression in alveolar epithelial cells

**Fig. 6:**
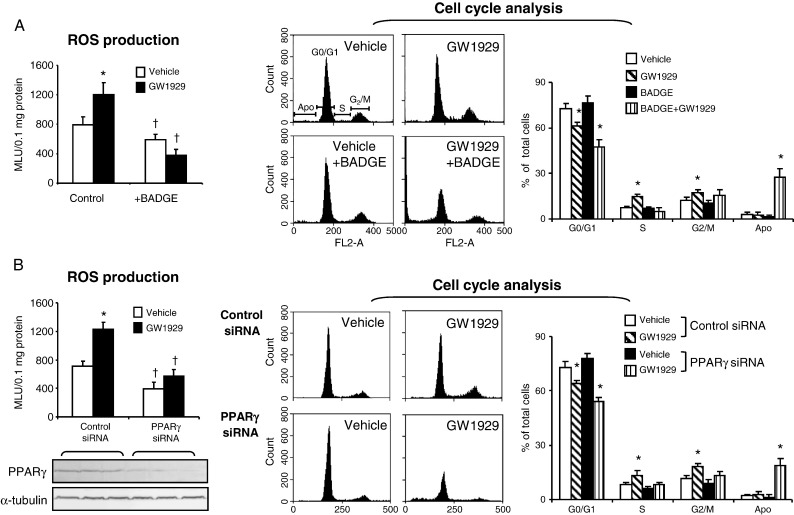
ROS production and cell-cycle analysis of isolated alveolar epithelial cells from wild-type mice. (A) The effects of PPARγ blockade by BADGE. (B) The effects of PPARγ knockdown by siRNA. Transfection efficiency was shown by Western blot. Left: NADPH-dependent O_2_^•−^ production detected by lucigenin chemiluminescence. *P < 0.05 for indicated values versus vehicle control values. † *P* < 0.05 for indicated values versus control values in the same treatment group. Right: Flow cytometry. *P < 0.05 for indicated values versus vehicle values in the same cell-cycle phase.

To find out if the effects of GW1929 on lung ROS production and cell-cycle progression were indeed on alveolar epithelial cells, we isolated alveolar epithelial cells by primary explant culture. The purity of the alveolar epithelial cells was confirmed, as they are ~ 95% positive for cytokeratin (an epithelial cell marker) and negative for CD31 and CD45 as detected by flow cytometry (Supplementary Fig. 2). There were ~ 11% of cells positive for vimentin (a fibroblast marker), which is well known to be weakly expressed by proliferating epithelial cells in culture. Cells were exposed either to vehicle or to GW1929 (5 μM) for 48 h in the presence or absence of a known PPARγ antagonist, BADGE (1 μM) [19], and examined for NADPH-dependent O_2_^•−^ production and cell-cycle progression (Fig. 6A). Compared to the vehicle controls, treatment of cells with GW1929 significantly increased the levels of O_2_^•−^ production (Fig. 6A, left) and promoted cell-cycle progression from G0/G1 phase to S and G2/M phase, i.e., the cell numbers in G0/G1 phase were significantly reduced, and those in S and G2/M phases were significantly increased (Fig. 6A, right). Blockade of PPARγ with BADGE reduced slightly but significantly the levels of ROS production without a significant effect on cell-cycle progression. However, when PPARγ was blocked by BADGE, treatment of cells with GW1929 reduced significantly the levels of ROS production, which was accompanied by cell detachment and death. Within the remaining adherent cells, ~ 30% were apoptotic (Fig. 6A, right).

The critical role of PPARγ in promoting alveolar epithelial cell ROS production and cell-cycle progression was further examined by knockdown of PPARγ using siRNA (Fig. 6B). The transfection efficiency was confirmed by Western blot (Fig. 6B, left bottom). In cells transfected with scrambled control siRNAs, GW1929 treatment significantly increased the O_2_^•−^ production (Fig. 6B, left) and promoted cell-cycle progression from G0/G1 phase into S and G2/M phases (Fig. 6B, right) compared to vehicle-treated control cells. Knockdown of PPARγ significantly decreased the levels of ROS production in vehicle-treated cells without significant effect on cell-cycle progression. However, when PPARγ was knocked down, GW1929 treatment failed to increase ROS production by cells, and this was accompanied by cell detachment and death. In the remaining adherent cells, ~ 20% were apoptotic (Fig. 6B, right).

### The effects of Nox2 siRNA or Nox2 knockout on GW1929-induced lung ROS production and cell-cycle progression

**Fig. 7:**
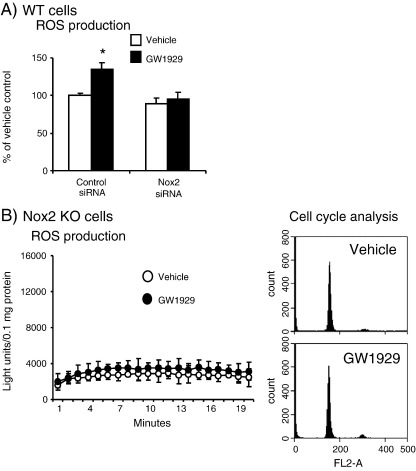
The effects of Nox2 siRNA or Nox2 KO on alveolar cell O_2_^•−^ production and cell-cycle progression. (A) Wild-type alveolar epithelial cells. (B) Nox2 KO alveolar epithelial cells. NADPH-dependent O_2_^•−^ production was measured by lucigenin chemiluminescence. Cell cycle progression was measured by flow cytometry. *P < 0.05 for indicated values versus control vehicle values; *n* = 3 independent cell isolations.

To confirm that Nox2 was indeed responsible for GW1929-induced O_2_^•−^ production by alveolar epithelial cells, we transfected wild-type cells with Nox2 siRNA and examined the levels of NADPH-dependent O_2_^•−^ production (Fig. 7A). In cells transfected with scrambled control siRNAs, GW1929 treatment significantly increased the ROS production. However, in cells transfected with Nox2 siRNA, GW1929 had no significant effect on cell O_2_^•−^ production. Finally, the critical roles of Nox2 in GW1929-induced O_2_^•−^ production and cell-cycle progression were confirmed using alveolar epithelial cells isolated from Nox2 KO mice (Fig. 7B). We found that Nox2 KO cells had a very low level of NADPH-dependent O_2_^•−^ production compared to wild-type cells (shown in Fig. 6A) and GW1929 treatment had no significant effect on the levels of O_2_^•−^ production by these cells. Accordingly, there was no significant difference in cell-cycle profiles between vehicle-treated and GW1929-treated Nox2 KO cells (Fig. 7B, right).

## Discussion

It is well known that PPARγ has a wide range of actions on glucose homeostasis and metabolism, and many exogenous PPARγ agonists have been used to treat type 2 diabetes and insulin resistance. However, PPARγ has many other actions on cellular biology, including cell proliferation, differentiation, and apoptosis, which may cause therapeutic complications such as fluid retention, cardiac hypertrophy and failure, and potential carcinogenicity [3]. The carcinogenicity issue is controversial because of discrepancies between studies. For example, most studies reported that PPARγ activation inhibited cancer cell proliferation and tumor growth, whereas others found that PPARγ activation potentiates tumorigenesis [20]. Different types of tissues respond differently to PPARγ activation and the mechanistic data are still missing to explain the mode of its involvement in tumor development. In this study we report, for the first time, that PPARγ activation through Nox2-derived ROS promotes cell-cycle progression in normal mouse lungs. We showed clearly, using middle-aged wild-type and Nox2 KO mice, that systemic administration of a specific PPARγ agonist (GW1929) increased the ROS production from the Nox2 enzyme specifically in the lung, and not in the heart, skeletal muscle, aorta, brain, or liver. Increased ROS levels were associated with increases in MAPK activation and the expression of PCNA and cyclin D1, two of the most important modulators directly involved in cell proliferation and cell-cycle progression. These effects were mainly due to PPARγ actions on alveolar epithelial cells as demonstrated by experiments using primary alveolar epithelial cells isolated from the wild-type mice and confirmed by blockade of PPARγ with BADGE (an identified PPARγ antagonist) [19] or knockdown of PPARγ using siRNA.

ROS have multiple effects on cellular function depending on the amount and subcellular location of the ROS generated. The cell cycle has been proposed to be a redox cycle [21]. Physiological changes in intracellular ROS levels are critical for cellular signaling and survival. However, prolonged oxidative stress or lack of oxidative stimulation causes cell apoptosis and death. In this study, we found that increased ROS production by Nox2 in response to PPARγ activation by GW1929 induced PCNA and cyclin D1 expression and promoted primary alveolar epithelial cell-cycle progression from G1 into S and G2/M phase. However, when ROS levels were low because of PPARγ blockade by BADGE or knockdown by siRNA, the cell cycle arrested. Moreover, when PPARγ was not available, GW1929 failed to increase cellular ROS production and caused cell death.

The mitogenic effects of ROS involve the modulation of redox-sensitive signaling molecules such as the MAPK superfamily, which lead to the activation of transcriptional factors and cell-cycle molecules, i.e., cyclins and cyclin-dependent kinases. MAPKs, in particular ERK1/2, have been shown to be involved in mediating PPARγ action in mouse myoblast cells and lung carcinoma cells [1,22]. In accordance with these previous reports, we found that PPARγ activation led to ERK1/2 and p38MAPK activation in the lung of normal mice, but not in the Nox2 knockout mice. Moreover, we found that PCNA and cyclin D1 were the downstream cell-cycle targets of ROS generated by Nox2 in response to PPARγ activation.

Cell cycle progression is tightly controlled by interactions between cyclins and cyclin-dependent kinases, and many of these cell-cycle molecules, such as cyclin D1, contain redox-sensitive cysteine residues and respond to the fluctuations in intracellular redox status that are linked to cell metabolism [21]. Nox2 has been found to be expressed constitutively in alveolar epithelial cells and plays important roles in the regulation of normal lung function and is involved in the pathogenesis of many lung diseases [11]. Previously, we have reported a critical role for O_2_^•−^ generated by Nox2 in cell-cycle regulation in endothelial cells [23]. In the current study, we found that Nox2 is involved in mediating PPARγ signaling in the lungs. Increases in O_2_^•−^ levels in response to PPARγ activation were not due to the changes in lung Cu/Zn SOD activity because inhibition of Cu/Zn SOD with or without a specific inhibitor, DDC, had no significant effect on the proportions of GW1929-induced ROS production in these samples [24]. Furthermore, the critical roles of Nox2-derived ROS in mediating PPARγ signaling were confirmed by (1) knocking down Nox2 using siRNA, (2) using cells isolated from Nox2 knockout mice, and (3) treating Nox2 knockout mice with GW1929. In these cases, GW1929 failed to increase ROS production or to promote cell-cycle progression. A putative PPRE (motif TGACCT) can be found in the Nox1 and Nox2 promoter regions. Interestingly, the PPRE in the Nox2 promoter is in an antisense orientation and is next to another PPRE-related motif (TGAAC) [25]. Further detailed investigation of PPARγ transcriptional regulation of Nox2 is required for understanding the mechanism.

It is also well documented that alveolar epithelial cells express Duox (Duox1 and Duox2), which generates mainly H_2_O_2_ in response to microorganism invasion or during epithelial differentiation [11]. However, different from Nox, Duox contains Ca^2+^-binding domains and its activation requires Ca^2+^ mobilization [11]. Therefore, Duox is unlikely to be the major source of PPARγ activation-induced O_2_^•−^ production in the lungs.

Although there is no evidence directly linking long-term PPARγ activation to lung cancer, patients suffering from type 2 diabetes have been found to have an elevated risk of cancer development, including lung carcinomas, regardless of their therapy [9]. PPARγ expression is highly up-regulated in lung cancers and has been suggested as a potential marker for lung cancer [26]. It is well known that PPARγ ligands have distinct activities between different cell types, between tumor cells derived from the same tissue, and between different tissues [1,3]. Cell proliferation and cell-cycle progression induced by thiazolidinediones (PPARγ agonists) have been reported in normal human bronchial epithelial cells [27], and specific knockout of PPARγ in the lung epithelium affected the postnatal lung development such that the mice had enlarged lung air spaces and decreased lung tissue resistance [7]. A PPARγ agonist has been found to increase lung angiogenesis [8]. It is therefore possible that long-term administration of PPARγ agonists may compromise pulmonary epithelial cell function and cell-cycle regulation [27].

There is controversy in the literature regarding the effects of PPARγ activation on lung epithelial cells depending on the type of cells used for the studies. Most previous studies were carried out on lung carcinoma cells or immortalized cell lines, or in animal models of growing lung cancers, and described PPARγ agonists as inhibitors of lung oxidative stress and cell-cycle progression [1,22]. However, mechanistic information and redox-signaling pathways of PPARγ in normal alveolar epithelial cells are still missing. In this study, we found that PPARγ activation by GW1929 promoted primary alveolar epithelial cell ROS production by Nox2 and cell-cycle progression. The discrepancy between ours and most previous studies might rest on the fundamental differences between healthy lungs and lung carcinomas and between primary normal alveolar epithelial cells and cancerous cells. On the other hand, the lung is at high risk for oxidative stress, as it interacts with various airborne oxidants. Therefore, PPARγ activation-induced Nox2 activation and ROS production may also represent a mechanism of adaption or protection from a diverse range of internal and external stimuli, which may be a beneficial response. Further investigation is needed to determine the role of PPARγ in the development of lung diseases.

In conclusion, we have reported for the first time that PPARγ is involved in the regulation of lung ROS production by Nox2 and the redox regulation of cell-cycle progression. PPARγ activation induces Nox2-dependent ROS production, which results in MAPK activation, PCNA and cyclin D1 up-regulation, and cell-cycle progression in alveolar epithelial cells. The mechanistic data from our study provide insights into PPARγ redox signaling and its effects on lung pathophysiology.

The following are the supplementary materials related to this article.

Supplemental Fig. 1 Original picture of Nox4 detection in wild-type lung homogenates by Western blot.

Supplemental Fig. 2 Characterization of isolated alveolar epithelial cells by flow cytometry.

## Figures and Tables

**Table 1:** Blood pressure and metabolic measurements.

Measure	Wild-type		Nox2 knockout	
	Vehicle	GW1929	Vehicle	GW1929
Body weight (g)	40.7 ± 0.8	42.6 ± 2.0	40.2 ± 4.2	38.3 ± 3.1
Systolic blood pressure (mm Hg)	140.7 ± 11.4	146.3 ± 15.7	139.2 ± 14.0	145.6 ± 16.3
Diastolic blood pressure (mm Hg)	110.8 ± 7.0	121.0 ± 13.0	113.5 ± 9.7	119.6 ± 19.5
Serum insulin (μg/L)	1.048 ± 0.67	1.122 ± 0.66	0.998 ± 0.98	0.745 ± 0.42
Fasting blood glucose (mM)	6.54 ± 0.45	6.07 ± 0.55	5.33 ± 0.40	5.57 ± 1.1
