# Supplementary material for: Nox2-derived ROS in PPARγ signaling and cell-cycle progression of lung alveolar epithelial cells
Source: Free Radic Biol Med. 2011 Aug 1;51(3-3):763–72. doi: 10.1016/j.freeradbiomed.2011.05.027 (PMC3157571; doi:10.1016/j.freeradbiomed.2011.05.027)
Supplement: Supplemental Fig. 1 — Original picture of Nox4 detection in wild-type lung homogenates by Western blot. [file mmc1.ppt]

## Slide 1
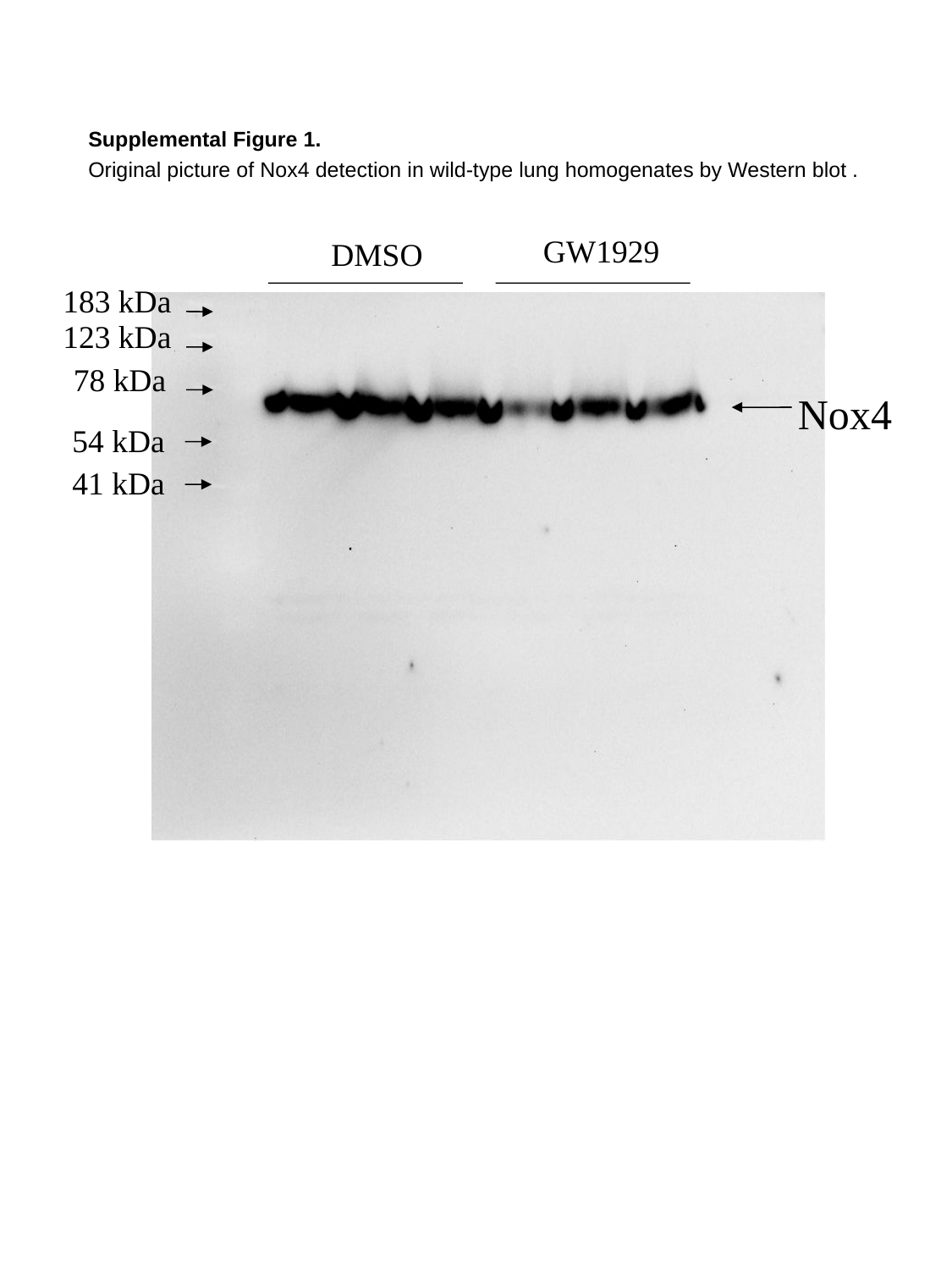

Supplemental Figure 1.
Original picture of Nox4 detection in wild-type lung homogenates by Western blot .
GW1929
DMSO
183 kDa
123 kDa
78 kDa
Nox4
54 kDa
41 kDa
