# Supplementary material for: Nox2-derived ROS in PPARγ signaling and cell-cycle progression of lung alveolar epithelial cells
Source: Free Radic Biol Med. 2011 Aug 1;51(3-3):763–72. doi: 10.1016/j.freeradbiomed.2011.05.027 (PMC3157571; doi:10.1016/j.freeradbiomed.2011.05.027)
Supplement: Supplemental Fig. 2 — Characterization of isolated alveolar epithelial cells by flow cytometry. [file mmc2.ppt]

## Slide 1
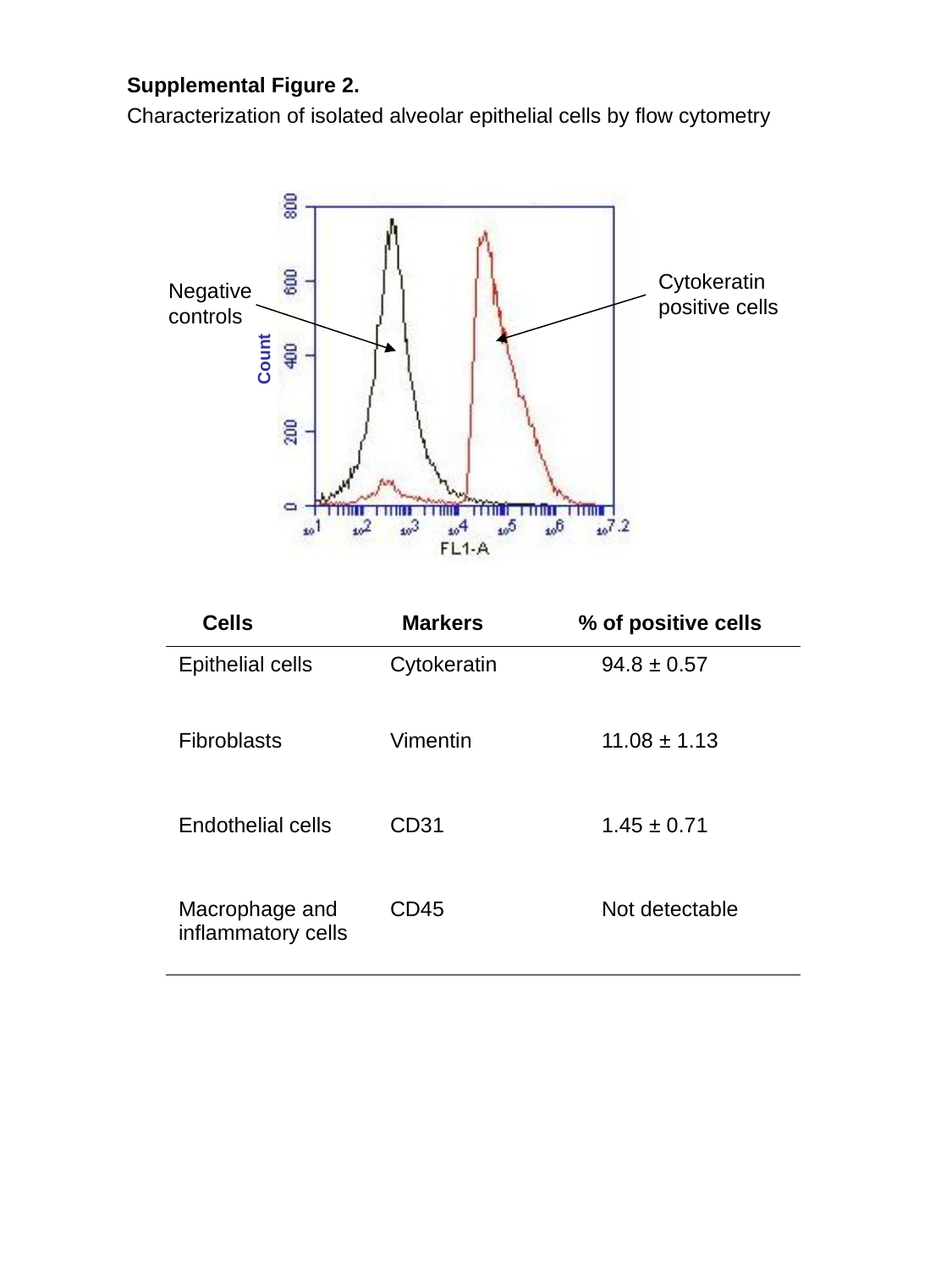

Supplemental Figure 2.
Characterization of isolated alveolar epithelial cells by flow cytometry
Cytokeratin
positive cells
Negative controls
Count
Markers
% of positive cells
Cells
| Epithelial cells | Cytokeratin | 94.8 ± 0.57 |
| --- | --- | --- |
| Fibroblasts | Vimentin | 11.08 ± 1.13 |
| Endothelial cells | CD31 | 1.45 ± 0.71 |
| Macrophage and inflammatory cells | CD45 | Not detectable |
